# Supplementary material for: Chromosome End Diversification in Sciarid Flies
Source: Cells. 2020 Nov 5;9(11):2425. doi: 10.3390/cells9112425 (PMC7694509; doi:10.3390/cells9112425)
Supplement: Supplementary file 1 [file cells-09-02425-s001.pdf]

cells

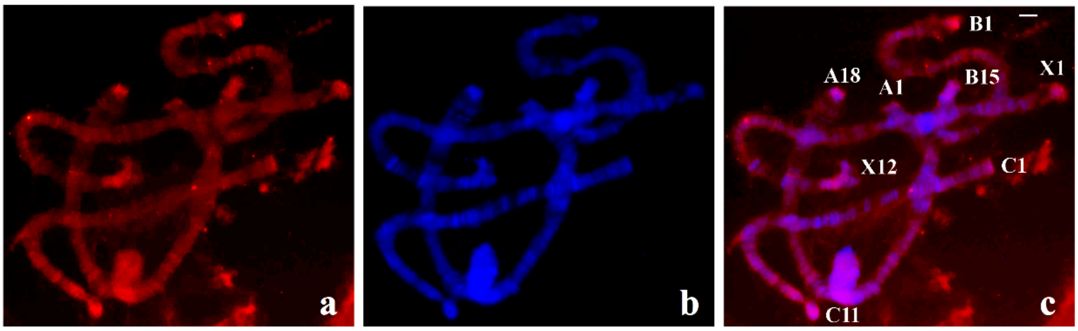

Supplementary Figure S1. (**a**) Polytene chromosomes of *R. americana* after in situ hybridization (red signal) using the probe made of DNA from the microdissected X-1 chromosome end of *T. pubescens*. (**b**) The same chromosomes stained with DAPI and (**c**) the corresponding merged signals. Bar =15 µm.

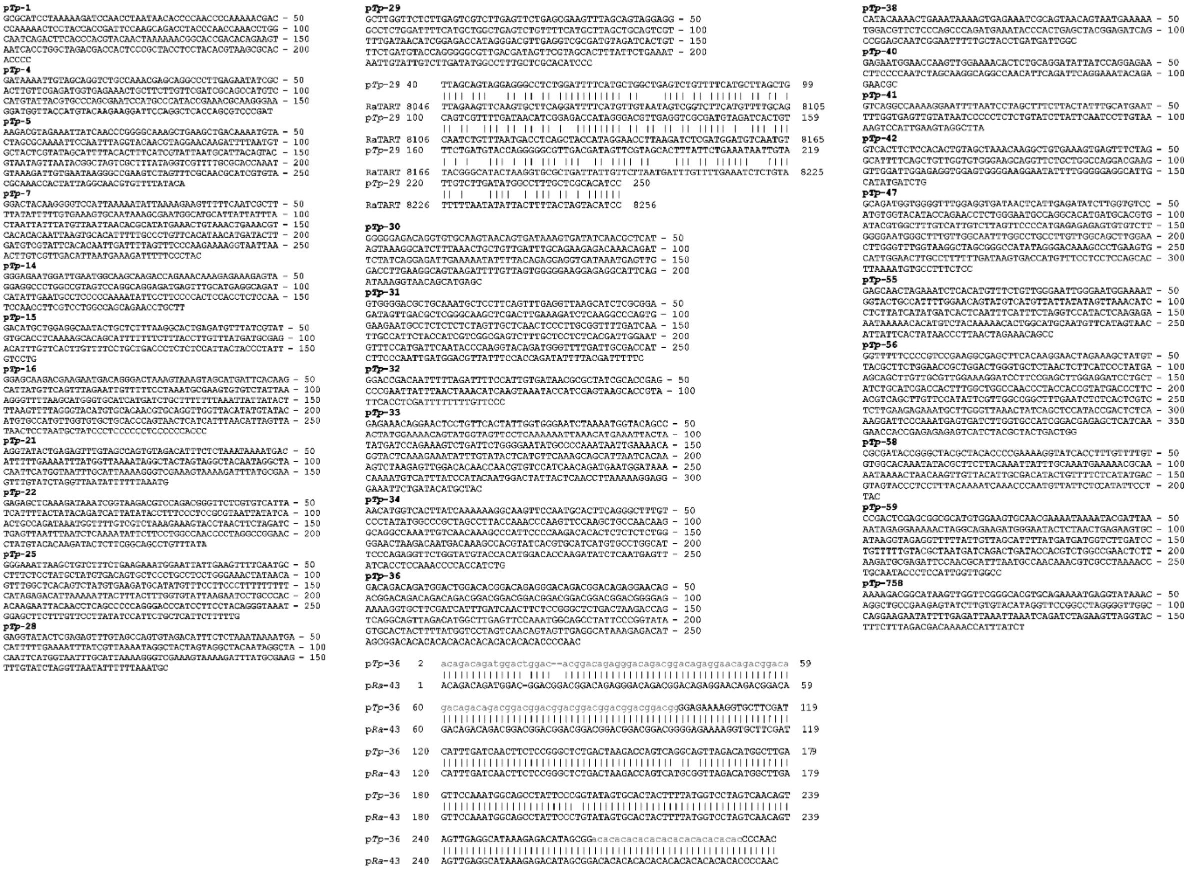

Supplementary Figure S2. Results of insert sequencing from the plasmid library made with DNA from the microdissected X-1 chromosome end of *T. pubescens*.

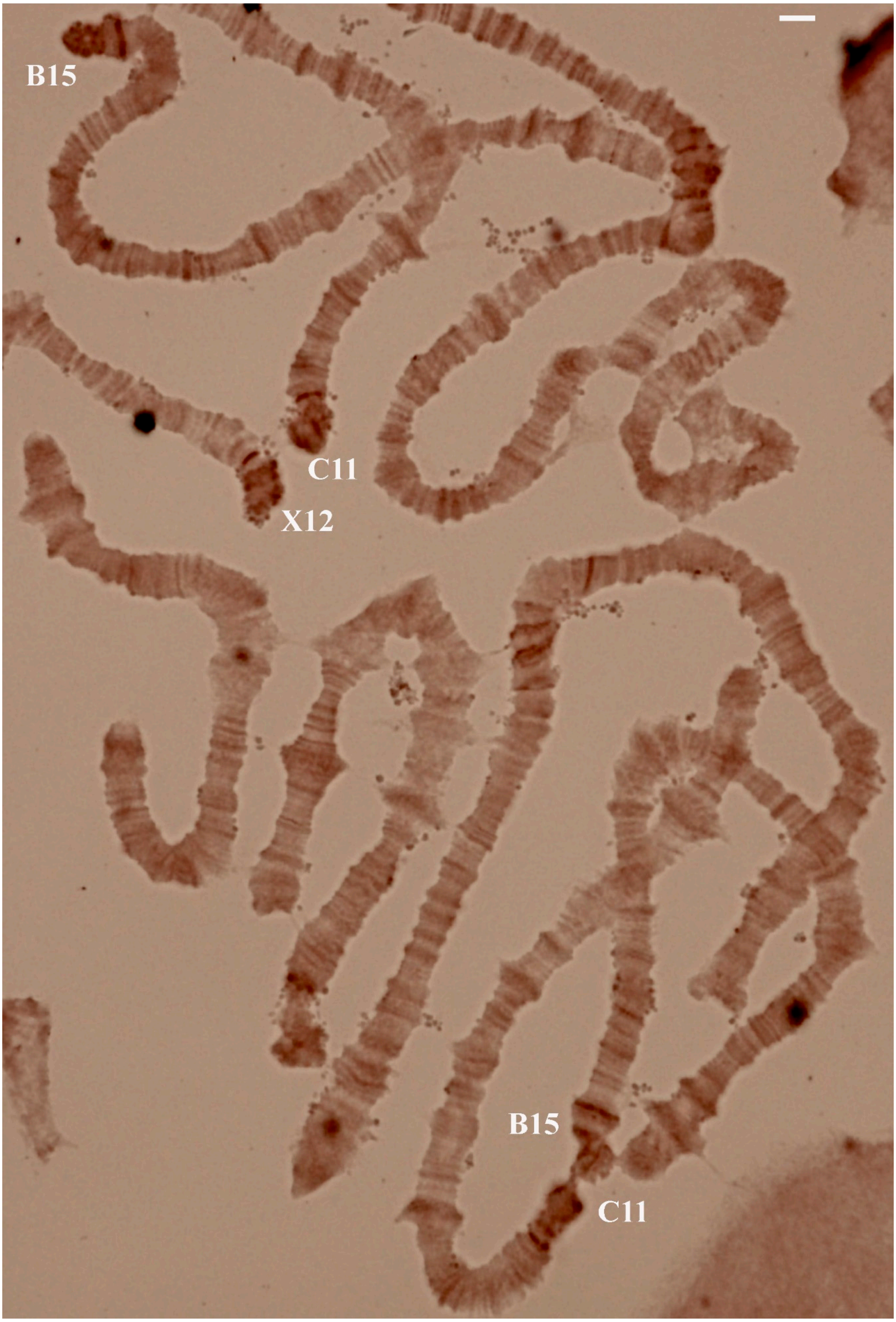

Supplementary Figure S3. Localization of phage 2 probe from the screening of the genomic library of *T. pubescens* in chromosomes of *R. americana*. Hybridization detection was made with alkaline

phosphatase and the chromosomes photographed under bright field. Heterochromatic ends identified in the figure correspond to the regions containing more intense hybridization signals.

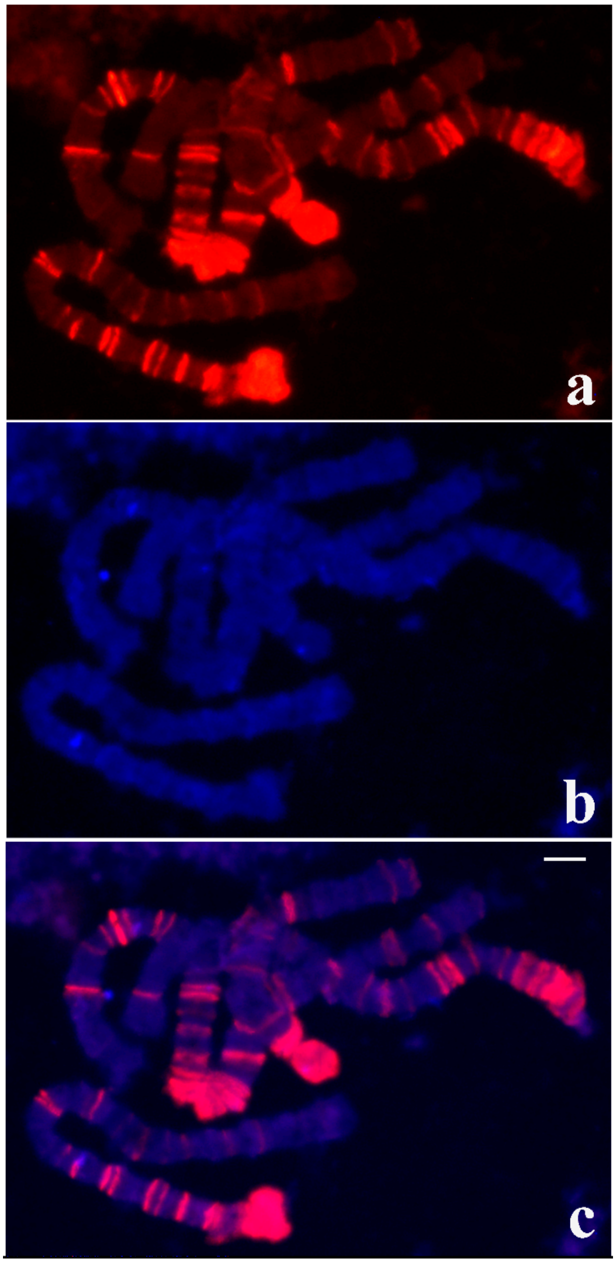

Supplementary Figure S4. Localization of p*Tp*-1 probe in *T. pubescens* chromosomes. (**a**) hybridization signals, (**b**) chromosomes stained with DAPI and (**c**) the merged hybridization and DAPI signals. Bar = 15 µm.

a

>pTp-2.1R

TGTCCCCGTTTCGAACGAGAGCGGTCGGAGGTTTTAATCGAAAACGGAGTAGTTCTGCATGCCGATAA

TG

TAGGGCGGAAATGTGCAGTGACGAGAAAGTGTGAAAGCGGTGGACAATGCAGTTGCGAAGATAATG

GGAA

GGCTGCAAAGTACATGGAGAGAGGAGCAGCGCAGCGAAGCAAATCGTCTGATGGTCTCTGATGGCGC

GTA

AGAGCGCAGCCCCTCCCCGAAGTAATGCTGTGAGGCGGTTCCGGGGAGACGAGGGCGTGGTGGCCAA
GGA
GTGAGGGTTTAGTCGGTAAGAATCCGACACTCTGGGTGCTAATTCCAGATGTCTATGAAGATTCCCTC
AA
CCTTGTAAAAAAAAAAAAAAAAAAAAAAAAAACACACACACACACACGTAAATTATTTCACTTA
TTTG
CTACTTTTTGTAGCAGTATTTACGGTCTTTGTTATCATGATTCGTGAAGTAAACACTGAAGCTCACTGTG
TACGTAATTTATTCCGACTCCCATAAAATCTGTCAATAAAGGAATAATTTTATAACAATGTACCATTCA
A
GACCTATTATGTTCTTCGACGTCCTGGAAATTGACAGATAATAAGGTAAACATTTCGCTTACAGTTTCA
A
ACTGGTCTGAAATACACTTAGGAAGCTTCTCGTGAAGTATCGTTTTAGGAAACTAGGGGAGACACTGG
TT
ACTCACCTTACAATGACTTTTAGTAAGCGATTATAAAACTACATCAAACACTTCATCTTAAATGTCACA
A
TAACTACACGTAAAAAGTACAATATGAATAAAATGATTTGCCTTCACGTTACTTCCTCACAATTAAAT
GG
GTCCTGGTAATGAACTAGTTGGTGATGAACCTATAGCCAGTGATGATCGTATAGCTCAACAACATGAC
AT
AGCGTATGACAATGCAGTTGACGAAGGTGACATTTATGAAGCTGACGAGAGGGCGATTTTTGCATTCG
TA
CTAGATTGGATTAAAAAATAAAAATTGGCATTCTGCTATTGGAGTCATGGGTTTAGGCGTTAAACATTT
A
TTAGATGTAATATGCTGTAGAGTTTTTATTGAAGTAATTGGTCNANAGGACTGGTGAACACCACTGTC
G
ATAACCGTTTGCATTGGATGTACAGAGAAAAGATCCAATTCTGATTTTACATTCATTTCATGAGCTTTG
T
TTATGTACGCCATTTTTAATTTGTAAAAAAAATCGTTATGTTTTTTCTTGTTGTTCTAGTAGTTGCCTTT
TTAGTCGTTACTGTTTTACGTTTCTTGATGTTTGCCCGTTGAGNGACTTTACTCGAATTGTGTTTGCTCT
ACTTTGGAAAGTTTTCTTGCTGTTTGGGTGANAAGTTTTTTCTTAAAATTGTNTTCTCAG

b

VP [Haematobia irritans densovirus]

Sequence ID: QCH41363.1Length: 687 Number of Matches: 1

Range 1: 4 to 64 GenPeptGraphics **Alignment statistics for match #1**

Score	Expect	Method	Identities	Positives	Gaps
61.2 bits(147)	6e-06	Compositional matrix adjust.	31/61(51%)	40/61(65%)	0/61(0%)

+3

Query 813 FTLLPHN*MGPGNELVGDEPIASDDRIAQQHDIAYDNAVDEGDIYEADERAIFAFVLDWI 992
FTL+ + +GPGN L EPI D IA++HDIAYD A + DIY+AD AI F D++
Sbjct 4 FTLIGYKYLGPGNSLDKGEPINKADSIAREHDIAYDRAKTKEDIYKADREAIIQFKDDFL 63

Query 993 K 995
K
Sbjct 64 K 64

Structural protein [Periplaneta fuliginosa densovirus]

Sequence ID: NP_051016.1Length: 242 Number of Matches: 1

Range 1: 154 to 223GenPeptGraphics Alignment statistics for match #1

Score	Expect	Method	Identities	Positives	GapsFrame
48.5 bits(114)	0.019	Compositional matrix adjust.	28/70(40%)	39/70(55%)	0/70(0%)

+3

Query 828 HN*MGPGNELVGDEPIASDDRIAQQHDIAYDNAVDEGDIYEADERAIFAFVLDWIKK*KL 1007

H+ +GPGN L +EP+ DD IA++HD AY NA D+ AD++AI F D+ K L

Sbjct 154 HHYLGPGNPLDNNEPVDRDDAIAEEHDKAYANAKSSIDVINADKKAIDHFSEDFEKNGNL 213

Query 1008 AFCYWSHGFR 1037

G +

Sbjct 214 HSLIGKTGLQ 223

Viral protein, partial [Helicoverpa armigera densovirus]

Sequence ID: AEI26261.1 Length: 448 Number of Matches: 1

Range 1: 8 to 63GenPeptGraphics Alignment statistics for match #1

Score	Expect	Method	Identities	Positives	GapsFrame
50.8 bits(120)	0.007	Compositional matrix adjust.	27/57(47%)	35/57(61%)	1/57(1%)

+3

Query 828 HN*MGPGNELVGDEPIASDDRIAQQHDIAYDNAVDEGDIYEADERAIFAFVLDWIKK 998

HN +GPG + EP+ DD IA++HD+AY NA DIY+AD+ A F D KK

Sbjct 8

HNYLGPGTKDFTKEPVDEDDEIARRHDLAYANATSHQDIYKADKEASKEF-FDSFKK 63

Structural protein VP [Human CSF-associated densovirus]

Sequence ID: ANG55948.1 Length: 840 Number of Matches: 1

Range 1: 243 to 302GenPeptGraphics Alignment statistics for match #1

Score	Expect	Method	Identities	Positives	GapsFrame
50.4 bits(119)	0.015	Compositional matrix adjust.	26/60(43%)	37/60(61%)	0/60(0%)

+3

Query 810 AFTLLPHN*MGPGNELVGDEPIASDDRIAQQHDIAYDNAVDEGDIYEADERAIFAFVLDW 989

+TL H +GPGN + + P+ DD IA++HD AY A ++ DI ADE+A F+ DW

Sbjct 243 GYTLPGHKFIGPGNPVDTEVPVDEDDAIAKEHDEAYATAKNQKDIAIADEQAADKFLSDW 302

Supplementary Figure S5. (**a**) Partial sequence of p*Tp*-2.1 plasmid. (**b**) Protein alignment results obtained by BLASTx.
